# Analysis of the *piggyBac* transposase reveals a functional nuclear targeting signal in the 94 c-terminal residues

**DOI:** 10.1186/1471-2199-9-72

**Published:** 2008-08-11

**Authors:** James H Keith, Tresa S Fraser, Malcolm J Fraser

**Affiliations:** 1 University of Notre Dame, Notre Dame, Indiana, USA

## Abstract

**Background:**

The *piggyBac* transposable element is a popular tool for germ-line transgenesis of eukaryotes. Despite this, little is known about the mechanism of transposition or the transposase (TPase) itself. A thorough understanding of just how *piggyBac* works may lead to more effective use of this important mobile element. A PSORTII analysis of the TPase amino acid sequence predicts a bipartite nuclear localization signal (NLS) near the c-terminus, just upstream of a putative ZnF (ZnF).

**Results:**

We fused the *piggyBac* TPase upstream of and in-frame with the enhanced yellow fluorescent protein (EYFP) in the *Drosophila melanogaster* inducible metallothionein protein. Using Drosophila Schneider 2 (S2) cells and the deep red fluorescent nuclear stain Draq5, we were able to track the pattern of *piggyBac* localization with a scanning confocal microscope 48 hours after induction with copper sulphate.

**Conclusion:**

Through n and c-terminal truncations, targeted internal deletions, and specific amino acid mutations of the *piggyBac* TPase open reading frame, we found that not only is the PSORTII-predicted NLS required for the TPase to enter the nucleus of S2 cells, but there are additional requirements for negatively charged amino acids a short length upstream of this region for nuclear localization.

## Background

*piggyBac *is a short repeat, target-site-specific transposable element originally isolated as mutational insertions within baculovirus genomes that originated from the infected TN-368 cells (*Trichoplusia ni*: Lepidopteran) [1]. This 2.4 kb transposable element is bounded by an asymmetric repeat configuration consisting of terminal 13 bp and sub-terminal 19 bp inverted repeats separated by either a 5' 3 bp or 3' 31 bp spacer [1]. The single *piggyBac *open reading frame is 1783 bp long, coding for a protein of 594 amino acids with a predicated mass of 68 kDa [1,2]. TPase catalyzed movement of *piggyBac *was originally demonstrated by utilizing the baculovirus genome in transfected *Spodoptera frugiperda *cell cultures as a target for the transposed DNA, and subsequently repeated using simple and rapid tests such plasmid excision assays [2] and interplasmid transposition assays which test for the removal of transposed DNA and its subsequent reinsertion into a different plasmid, respectively. The tests can be carried out in both transfected insect cells and microinjected insect embryos [3].

The *piggyBac *element has several properties that make it an ideal tool for transgenesis, including site-specific integration and excision [2], proven large carrying capacity [4], controllable remobilization [5], and the ability to insert in heterochromatin and euchromatin throughout a genome, in both regulatory and coding regions, greatly facilitating enhancer trapping and random mutagenesis studies [5-7]. This is not to say that all of these properties are shared in all hosts for which they have been assayed. It should be noted that despite the function of *piggyBac *in the cells of *Spodoptera frugiperda *[8], attempts to transform the species itself have yet to be successful [9]. Simple plasmid-based mobility assays have also shown *piggyBac *to be active in human and other primate cells [4,10], in *Zea maize *cells [11], in *Saccharomyces cerevisiae *[12], and in the embryos of *Aedes triseriatus *[13], *Heliothis virescens *[14], and *Danio rerio *[10]. Of the species amenable to *piggyBac*-mediated germ-line or strain transformation, namely, *Plasmodium falciparum *[15], *Mus musculus *[4], *Tribolium castaneum *[5], *Anopheles gambiae *[16], *Ceratitis capita *[17], *Drosophila melanogaster *[18], *Bactrocera dorsalis *[19], *Musca domestica *[20], *Lucilia cuprina *[21], *Bicyclus anynana *[22], *Aedes aegypti *[23,24], *Anopheles albimanus *[25], *Anopheles stephensi *[26], *Bombyx mori *[27], *Athalia rosae *[28], *Drosophila willistoni *[29], *Pectinophora gossypiella *[30], *Anastrepha suspensa *[31], *Aedes fluviatilis *[32], *Harmonia axyridis *[33], and the human blood fluke *Schistosoma mansoni *[34], remobilization assays have only been attempted for *Aedes aegypti *[35], which was unsuccessful, and *Tribolium castaneum *[5], and *Drosophila melanogaster *[6], which both demonstrated remobilization function. In cases of straight transgene introduction, for example foreign protein production by silkworms, or RNAi studies, stable germ-line transformation is preferred. However, others situations, such as enhancer trapping and saturation mutagenesis, remobilization is desired. It is for these reasons *piggyBac *is proving a valuable tool for functional genomics in *D. melanogaster *[6] and quickly becoming the transposon of choice for germ line transformation [36].

The *piggyBac *TPase is the archetype of a family of related sequences [37] identified from both computer predictions and EST libraries in a diverse array of organisms such as *Takifugu rubripes*, *Xenopus*, *Daphnia*, and even *Homo sapiens *[38]. At present, five *piggyBac *transposable element derived (PGBD) genes, some with multiple isoforms, have been identified among human cDNA clones (Genbank acc#: D88259, CR623168, AK074682, AK094816, and CR597281, respectively) [37]. PGBD3, isolated from human testis cDNA (Genbank acc#: BC034479), overlaps with the excision repair cross-complementing 6 gene (ERCC6), which codes for the cockayne syndrome B protein, CSB [39]. The first 465 residues of PGBD3 and ERCC6 (1061 and 1493 amino acids, respectively) are identical, and occur in the same place on the genome. Cockayne syndrome is a devastating autosomal recessive disease marked by premature physical aging, loss of hair, UV hypersensitivity, and mental retardation. Other signs include severe tooth decay, a high predisposition for a number of cancers, osteoporosis, demyelination of nervous tissue, calcification of the cortex and basal ganglia, and neuronal loss [40].

The size of the *piggyBac *family, its wide utility as a transgene vector, and the patterns of *piggyBac *related protein expression in human tissues warrant a deeper investigation into the function of this obviously critical family of proteins. Through stepwise mutagenesis we can identify functional and catalytic domains for the TPase, and gain a better understanding of the functional properties of other members of the *piggyBac *family.

TPase catalyzed integration and excision occurs within the eukaryotic nucleus, necessitating either diffusion or transport of the protein across the nuclear envelope through the nuclear pore complexes (NPC). While proteins below a size threshold of 40–60 kDa can passively diffuse [41] through these pores, those of greater mass must be actively transported through pore complexes by nuclear import proteins [42]. Actively transported proteins require one or more nuclear localization signals (NLSs) that facilitate their interaction, either directly or indirectly, with nuclear transport proteins [43]. However, *piggyBac *may also reside in the nucleus using a nuclear retention signal. In this scenario, *piggyBac *avoids the requirement for active nuclear transport and could only enter the nucleus during mitosis when the nuclear envelope is not present. While nobody has yet explored the possibility that transposition may only occur during mitosis, and an NLS is not needed, other TPases have already been shown to have NLSs [44-50]. Since the *piggyBac *TPase has a demonstrated mass of nearly 68 kDa [51], there is no selective pressure for a nuclear retention signal in the absence of active transport as TPase cannot passively diffuse out of the nucleus once entered. We presume that if it is indeed active in the presence of a nuclear envelope, it requires active nuclear transport and therefore may contain a NLS. We therefore find reasonable cause to suspect *piggyBac *possesses an active NLS as well.

The mechanism for nuclear localization is highly conserved among eukaryotes. With the exception of a few specialized NLSs [52], a cell can recognize the NLS of exogenous proteins from highly divergent organisms [43]. Of those NLSs that have been identified, the two most widespread and well characterized are the classic bipartite and monopartite NLS [53,54]. Both of these signals rely on a tract of negatively charged amino acids that are essential for interaction with nuclear importin receptors. The wide host mobility for *piggyBac *suggests its TPase possesses a conserved NLS that conforms to at least one of the classical types of motifs and can operate in a large variety of cells.

Sarkar et al. indicate a PSORTII [55] analysis of the *piggyBac *TPase predicts a bipartite NLS that falls within a twenty-one amino acid stretch ('PVMKKRTYCTYCPSKIRRKAN') of the C-terminus including residues 551 through 571 [37]. This region of the TPase, in fact, contains four patterns matching characterized NLSs.

In this report we define the *piggyBac *NLS by constructing a series of *piggyBac *truncations and deletions fused in-frame and upstream of the fluorescent protein EYFP and comparing their nuclear localizing properties to that of a full length TPase-EYFP fusion in transfected *Drosophila *S2 cells. Using the PSORTII prediction as a starting point, we demonstrate that the regions of the TPase responsible for nuclear localization are located within the carboxy terminal 94 amino acids. Deletion of the PSORTII-predicted bipartite NLS, residues 551–571, eliminates nuclear targeting of the TPase-EYFP fusion protein. However, this sequence does not act as a NLS when placed at the amino-terminus of EYFP. The minimum deletion fragment of the *piggyBac *TPase required for nuclear localization of the EYFP protein includes the last 94 amino acids (501–594). Additional mutation analyses of *piggyBac *TPase-EYFP fusions further refine the NLS to within amino acids 501–571.

Point mutation analysis identifies at least three individual amino acids located a short distance upstream of the predicted NLS that are essential for nuclear import, but like the predicted NLS, are alone insufficient for nuclear localization. Together these data establish that while the predicted NLS alone is too short to form a recognizable active domain, in its native context within the TPase protein it functions in the translocation of the protein to the nuclear compartment.

## Results

### Full Length *piggyBac*

A PSORTII analysis of the predicted amino acid sequence for the *piggyBac *TPase identified NLS patterns between residues 551 and 571 that matched two known consensus signals. The first identified sequence, located at amino acids 554 through 571, is a region that is similar to the bipartite NLS originally defined for *Xenopus *nucleoplasmin [56] that is composed of 2 basic regions separated by a non-specific 10 residue spacer. This particular region of the TPase is so concentrated with basic amino acids that the bipartite consensus match can begin at either amino acid 554 or 555. In fact, the presence of basic residues in this region is so ubiquitous that, in addition to the bipartite signal, two regions consistent with the requirements for a monopartite NLS can be found in the same stretch: 'PVMKKRT' and 'PSKIRRK' at positions 551–557 and 563–569, respectively. These sequences resemble the monopartite signal exemplified by the SV40 large T antigen, which is defined as a proline followed by a basic region containing either arginine or lysine in 3 out of 4 residues, and within 3 residues of the original proline [54]. This result indicates that *piggyBac *has up to four possible classic NLS patterns in this short 21 amino acid region.

### Experimental identification of nuclear localization signals

**Figure 1:**
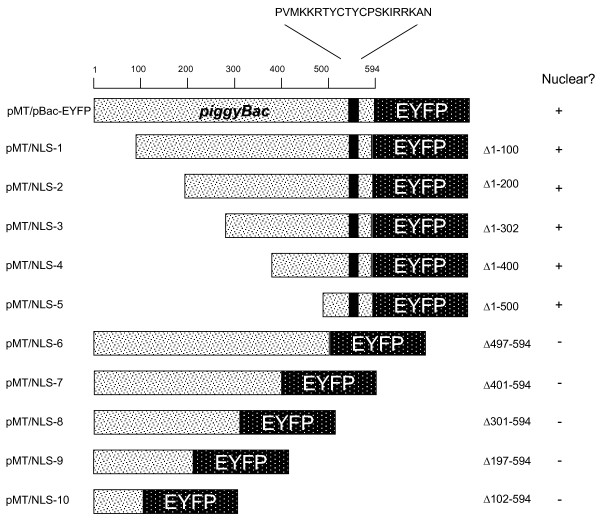
**piggyBac truncations**. The *piggyBac *TPase is shown as an N-terminal fusion to the enhanced yellow fluorescent protein (EYFP). The PSORTII-predicted NLS region is indicated by solid black. The name of each vector is to the left of the visual diagram with the actual changes made listed to the right of the diagram. The observed nuclear localization pattern is indicated in the right column. Sizes and distances are not necessarily to scale. Numbers represent amino acid positions with respect to the *piggyBac *start codon.

**Figure 2:**
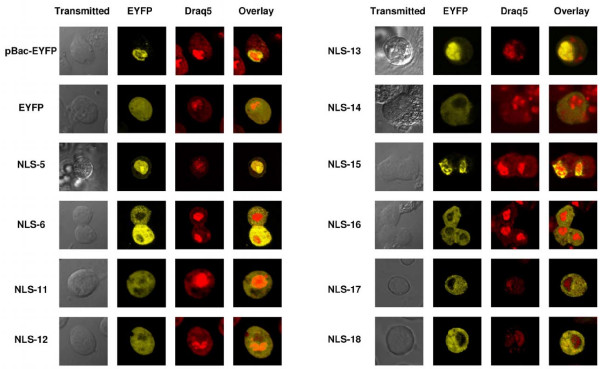
**Confocal microscopy**. Confocal microscope images for vectors described in the text. Vector names and their corresponding images are shown. The first column is a transmitted black and white image of the cell. The second column shows EYFP fluorescence pattern observed as a fusion protein with the *piggyBac* TPase. The third column is the nuclear stain Draq5 while the fourth column is an overlay of the EYFP fluorescence and Draq5 stain. All microscopy work was performed approximately 48 hours post induction. All images are the result of 6 lines averages performed by the imaging software. Each image is zoomed and cropped on the cell or cells of interest but all remain otherwise unenhanced and unaltered.

To obtain representative examples of what to expect with a nuclear localizing protein, and a diffuse protein, we first imaged the full *piggyBac *protein fused to EYFP, and the EYFP protein alone. Confocal imaging confirmed nuclear localization of the 96.5 kDa full length *piggyBac *TPase-EYFP fusion protein, coded by pMT/pBac-EYFP (fig. 1; fig. 2). The nucleus was readily evident in each picture, marked by the red emitting nuclear stain, Draq5. Yellow fluorescence was entirely absent from the cytoplasm and concentrated in the nucleus, which was visible by staining with Draq5. The 96.5 kDa pBac-EYFP fusion protein was well over the molecular weight threshold for passive diffusion of proteins into the nucleus, suggesting active nuclear transport was required. The distribution pattern observed for the pBac-EYFP product was distinctly different from that of the 28 kDa EYFP non-fusion protein control which yielded an evenly dispersed fluorescence in both cytoplasmic and nuclear compartments consistent with passive diffusion into and out of the nucleus (fig. 2). These results confirm an active nuclear localizing capability for the *piggyBac *TPase that facilitates nuclear import of proteins beyond the passive diffusion limit of 40–60 kDa.

### Truncation mutation analysis

We constructed both amino-terminal and carboxy-terminal deletion series for the *piggyBac *TPase to experimentally verify the location of a functional NLS within the 1782 bp *piggyBac *TPase open reading frame. We deleted *piggyBac *from either side in roughly 300 bp increments (fig. 1: pMT/NLS-1 through pMT/NLS-10) in two separate series of deletions. Each of these deletion series were fused upstream and in-frame with EYFP, and positioned for expression within the pMT vector.

The compartmentalization pattern for each expressed TPase truncation-EYFP fusion protein from either the N-terminal or C-terminal series was observed following transient expression of transfected S2 cells using confocal microscopy. Cells transfected with vectors expressing fusion proteins that retained the 94 carboxy-terminal amino acids of *piggyBac *exhibited yellow fluorescence that concentrated within the nucleus, while no significant nuclear localization was evident for EYFP fusions that did not contain these 94 carboxy-terminal amino acids. The smallest contiguous TPase fragment sufficient to yield distinct nuclear localization activity was the c-terminal 94 amino acid sequence expressed in pMT/NLS-5 (Δ1–500; fig. 2), while the largest TPase fusion of the C-terminal deletion series that failed to localize to the nucleus was pMT/NLS-6 (Δ497–594; fig. 2). The difference in localization patterns between the diffuse EYFP-only protein and the larger, nuclear-concentrated pMT/NLS-5 expressed protein was plainly visible. These results demonstrated that the nuclear localization signal must be located downstream of amino acid 500.

### Analysis of the carboxy-terminus

**Figure 3:**
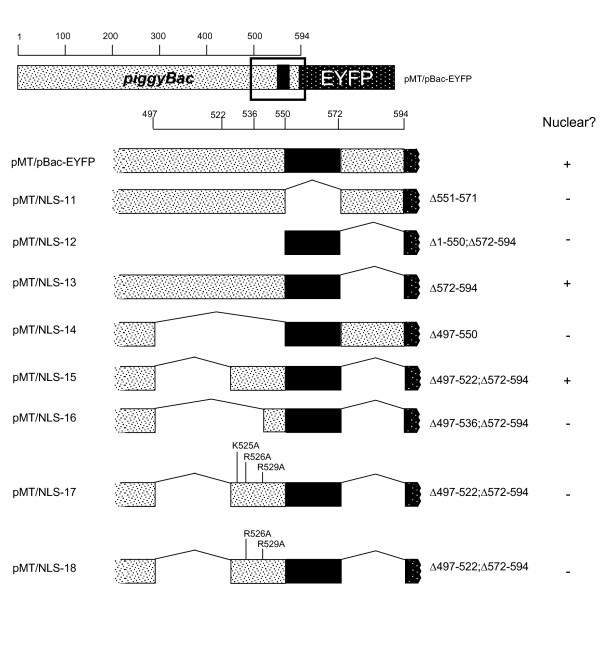
***piggyBac* mutation and truncation refinements**. Vectors used in the investigation of the nuclear localization pattern of *piggyBac* in and around the PSORTII-predicted NLS. Deletions are represented by bridged lines. Mutations are specifically indicated. The name of each vector is to the left of the visual diagram with the actual changes made listed to the right of the diagram. The observed nuclear localization pattern is indicated in the right column. Sizes and distances are not necessarily to scale. Numbers represent amino acid positions with respect to the *piggyBac *start codon.

The N-terminal and C-terminal truncations provided evidence that the carboxy terminal 94 amino acids of the *piggyBac *open reading frame were both necessary and sufficient to cause the nuclear localization of *piggyBac*. This sequence included the PSORTII-predicted NLS. We analyzed this region in detail to more accurately define the boundaries and function of the predicted *piggyBac *NLS. We constructed an in-frame fusion of the NLS-deletion upstream of the EYFP ORF to create pMT/NLS-11 (Δ551–571; fig. 3). Deletion of the entire PSORTII-predicted NLS eliminated expressed fluorescence from the nucleus of S2 cells (fig. 2) which confirmed the necessity of the PSORTII-predicted segment for nuclear localization. Interestingly, this fusion protein appeared to aggregate, forming small but distinct foci in the cytoplasm when viewed under higher magnifications. This aggregation differed significantly from the distribution obtained with the expressed EYFP control protein, which exhibited a diffused, homogenous fluorescence throughout both the nucleus and cytoplasm.

Next, we directly investigated the functionality of solely the PSORTII-predicted *piggyBac *NLS by fusing this short encoding segment between amino acids 551 and 571, inclusive, to EYFP to yield pMT/NLS- 12 (Δ1–550, Δ572–594; fig. 3). Although the molecular weight of the protein (28 kDa) was below the 40–60 kDa threshold for passive diffusion into the nucleus, the resulting protein was observed in both the nucleus and the cytoplasm (fig. 2), clearly different from pMT/pBac-EYFP. The failure of this fusion protein to concentrate solely in the nucleus indicated an inability of these residues to form a functional NLS domain, suggesting the function of this sequence is context-dependent.

### Importance of sequences flanking the NLS

Since fusion of TPase amino acids 551 through 571 to the N-terminus of EYFP did not allow direct confirmation of a NLS function for the PSORTII-predicted sequences, additional flanking amino acids likely contribute to the activity of this sequence, most likely through facilitation of proper folding. To confirm this hypothesis we constructed two TPase deletion mutations that omitted amino acids either upstream or downstream of the predicted NLS by PCR amplification of the pMT/pBac-EYFP plasmid using inverse-facing primers bounding the area to be deleted. Deletion mutation pMT/NLS-13 (Δ572–594; fig. 3) contained all the amino acids upstream of the predicted NLS. The pattern of fluorescence obtained with this deletion-fusion (fig. 2) was indistinguishable from that of the full length *piggyBac*-EYFP fusion protein, demonstrating that amino acids downstream of the predicted NLS are dispensable for efficient nuclear trafficking.

A second deletion-fusion, pMT/NLS-14 (Δ497–550; fig. 3), removed 54 residues upstream of the predicted NLS. The pMT/NLS-14 fusion protein (fig. 2) remained dispersed in the cytoplasm, demonstrating that the 54 amino acid sequence upstream of the NLS is likely involved in the proper presentation or functioning of the NLS domain.

Two additional deletion fusions in this 50 amino acid flanking sequence were also examined for possible contributions to the nuclear localization activity. The specific boundaries of the deletion constructs pMT/NLS-15 and pMT/NLS-16 were chosen based upon the presence of a proline residue at positions 522 and 537, respectively. Deletion fusions pMT/NLS-15 (Δ497–522, Δ572–594; fig. 3) and pMT/NLS-16 (Δ497–536, Δ572–594; fig. 3) were created by deleting portions of the *piggyBac *open reading frame between amino acid 497 and either proline 522 or proline 537, inclusive, utilizing the deletion plasmid, pMT/NLS-13 as the template. pMT/NLS-15 trafficked efficiently to the nucleus (fig. 2) while the fusion protein lacking the more lengthy segment, pMT/NLS-16, remained confined to the cytoplasm (fig 3). We emphasize that both of these fusion proteins had predicted masses well over the size threshold required for passive diffusion into the nucleus. Taken as a pair, the localization patterns of these two deletion proteins could be interpreted to indicate the NLS is between amino acids 523 and 535. However, pMT/NLS-11 also fails to enter the nucleus, suggesting that both these regions are required for nuclear localization. These results identified the segment of *piggyBac *required for proper presentation of the predicted NLS as contained somewhere between amino acids proline 522 and glutamic acid 550.

### Importance of basic amino acids proximal to the predicted NLS

The inability of the isolated TPase PSORTII-predicted NLS motif to cause nuclear localization suggested a role for the adjacent amino acids in the formation of a functional nuclear localization motif. Our deletion plasmids pMT/NLS-15 and pMT/NLS-16 confirmed the requirement for upstream amino acids. Investigation of the area between proline 522 and glutamic acid 550 revealed three basic amino acids K525, R526, and R529. These basic amino acids lie among a stretch of largely neutral residues.

Substitution of these residues with neutral amino acids would reveal any specific requirement for these in the nuclear localization of *piggyBac*. Two plasmids were created: pMT/NLS-17 (Δ497–522, Δ572–594, K525A, R526A, R529A; fig. 3), and pMT/NLS-18 (Δ497–522, Δ572–594, R526A, R529A; fig. 3). Simple replacement of these amino acids with uncharged residues disrupted the nuclear localization activity of fusion proteins that were otherwise trafficked to the nucleus, including those containing the predicted NLS (fig. 2). The altered fluorescence patterns for pMT/NLS-17 and pMT.NLS-18 reveals that while the bipartite signal may contribute the core nuclear localization activity to *piggyBac *TPase, additional flanking amino acids are somehow involved in the proper function of this signal.

## Discussion

Eukaryotic proteins that bind with or interact with DNA must be capable of entering the nuclear compartment. NLSs have been identified in several eukaryotic TPases including *Hermes *of *Musca domestica *[44], *mariner *of *Drosophila mauritania *[45], *BmTc1 *of *Bombyx mori *[46], *Mu *[47] and *Activator *[48] of *Zea maize*, *Tag1 *of *Arabidopsis Thaliana *[49], and the reconstructed salmonid transposon, *Sleeping Beauty *[50]. Previous studies have demonstrated the nuclear localization capacity of *Minos *of *D. hydei *[57]. Analysis of *Minos *by PSORTII predicts 4 separate amino acid sequences consistent with standard patterns. These are monopartite signals: 'PRDKRQL', 'KKKR', and 'PKRVKCV' at amino acid positions 67, 130, and 325 respectively and a bipartite signal, 'RKRSETYHKDCLKRTTK', at 172. Many prokaryotic recombinases and integrases exhibit enhanced activity in eukaryotic cells when they are linked with eukaryotic nuclear importation signal sequences. For example, recombination activity of the φ C31-integrase is enhanced in eukaryotic cells when the SV40 T-antigen archetypical NLS is fused to the carboxy terminus [58]. Because the *piggyBac *TPase is larger than the threshold size for passive diffusion it also must be actively targeted to the nucleus to be effective in target site recognition and transposition.

A PSORTII examination of the *piggyBac *TPase sequence predicted multiple mono- and bi-partite NLSs. The classic pat4 monopartite signal pattern is composed of three or four basic residues (K or R) followed by a H or P. Additionally, the monopartite signal can adhere to the pat7 pattern, having a P residue followed closely by a four residue stretch that contains at least three basic amino acids [54]. The bipartite signal follows a somewhat more defined consensus pattern with two basic amino acids followed by a ten residue spacer that connects to at least three out of five basic amino acids [56]. There is considerable variability in the ten residue spacer, allowing for a number of different motifs to be located within the bipartite NLS signal. Our data cannot rule out that either or both of the predicted monopartite signals are the true NLSs of the *piggyBac *TPase each with a requirement for the upstream basic amino acids for proper function.

The NLSs and nucleic acid binding domains of most proteins that exhibit both activities either overlap or are located immediately adjacent to each other [53]. This frequent overlap appears to result from co-evolution of the DNA interacting domain and nuclear localization signal for a given protein [59]. Several examples of overlap or close proximity between the two signals have been characterized [60]. In some cases the NLS may be too short to form an independent functional domain and may have additional requirements for adjacent residues to present a successful secondary structure for nuclear targeting. For example, the bipartite NLS of the human androgen receptor is fully dependent on the presence of the overlapping ZnF, which itself is responsible for DNA binding [61]. Cokol and colleagues (2000) analyzed publicly available protein motif information and concluded that for 90% of proteins in which both the DNA binding domain and NLS are known, these signals overlap. The flexibility of the ten residue spacer in the bipartite signal allows different DNA sequences to be targeted while preserving the underlying NLS pattern and function.

In fact, the location of the predicted bipartite NLS and the second predicted monopartite NLS of the *piggyBac *TPase overlap a ZnF motif 'CTYCPSKIRRKANASCKKCKKVICREHNIDMCQSCF' found at the very C-terminus of *piggyBac *TPase starting at residue 559. This ZnF is a novel match for the well-known RING-finger motif which, in the case of *piggyBac *TPase, starts in the spacing region of the bipartite signal and extends downstream to the end of the TPase. ZnFs are classically implicated in the DNA binding, while the RING-finger variant is more apt to be part of a protein-protein domain, a function that *piggyBac *would require either by itself or through interacting host factors in order to carry out transposition [62]. Previous work by our lab with western blots, co-immunoprecipitation, and the yeast two-hybrid system suggests a multimerization capacity of the TPase (unpublished). For instance, *piggyBac *has a proven ability to catalyze the transposition of a wide range of load sizes, implying that domains which interact with the *piggyBac *ITRs are not at a fixed distance relative to each other. Additionally, when used in a cartridge with one upstream ITR and a choice of either a proximal or a distal downstream ITR, *piggyBac *shows no particular preference for either ITR [51].

Deletion of the PSORTII-predicted bipartite NLS, located between amino acids 551 and 571, inclusive, eliminates nuclear targeting of the *piggyBac *TPase-EYFP fusion protein. However, addition of this same sequence at the amino-terminus of EYFP is insufficient to provide nuclear targeting. Fusion of a series of systematic deletions from both the carboxy and amino termini of the *piggyBac *TPase upstream of the marker protein EYFP allows us to define the minimum sequence sufficient for nuclear trafficking as the carboxy-terminal 94 residues. In addition, deletion of the last 23 amino acids of the *piggyBac *open reading frame, encompassing everything downstream of the bipartite NLS, demonstrates this region is unnecessary for nuclear localization. The fact that *piggyBac *is active in a wide range of host cells and species would indicate that any NLS it possesses is readily recognized by conserved nuclear importing machinery. We find no logical reason to suspect that an NLS displaying such a wide tropism would be any less conserved. We therefore conclude a functional NLS is contained within the 71 amino acids from 501 to 571, and in light of the wide activity of *piggyBac*, the active NLS is most likely one or more of the 4 common patterns predicted by PSORTII.

Our results also demonstrate that a segment of the TPase upstream of the predicted bipartite NLS is also essential for nuclear localization. We therefore attempted to define the involvement of these upstream sequences using point directed mutation analysis and further deletions.

The amino acid proline breaks the periodic structure of α-helices and β-sheets, dividing the structure of a protein from one functional domain to the next [63]. If the NLS of the *piggyBac *TPase lies within a larger conformational domain, then the start of such a domain may be indicated by a proline. Examination of prolines located upstream from the predicted bipartite signal for their possible involvement in delineating regions responsible for the proper presentation of the *piggyBac *NLS defined a smaller region comprised of amino acids 522 through 571 that is sufficient for nuclear localization. This region includes the predicted bipartite NLS and the 29 amino acids immediately upstream. Nuclear localization was unaffected by deletions upstream of proline-522, but removal of the residues between proline-522 and proline-537 completely abolished nuclear localization. However, these data alone cannot rule out an alternate interpretation that all four PSORTII-predicted NLSs are, in fact, necessary but nonfunctional, and that the upstream flanking basic amino acids constitute the true NLS by interacting in a novel manner with conserved importin machinery.

Alteration of the basic amino acids between proline-522 and proline-537 confirmed their importance in nuclear trafficking. The changes K525A;R526A;R529A and R526A;R529A each prevented the EYFP fusion proteins from entering the nucleus. Therefore, these arginines are somehow involved in the formation of a functional nuclear localizing domain within the context of a pBac-EYFP fusion. This requirement for proximal amino acids for the function of an NLS is not without precedent [61].

## Conclusion

We conclude from these findings that the *piggyBac *TPase has a functional NLS located between amino acids 551 and 571. However, our deletion and mutation constructs do not allow for a complete examination of the functionality of the monopartite signals either alone or in tandem, separate from the predicted bipartite NLS. Some NLSs function with non-native proteins when they are simply appended to the C-terminus [58], and some require flanking amino acids from their native context to retain nuclear import activity [61]. This short segment of amino acids in the *piggyBac *TPase is most likely the critical component of the nuclear localization function through its binding, either directly or through an adapter molecule, to a member of the importin family of proteins.

We have demonstrated a requirement for the presence of at least two basic amino acids located proximally upstream of the predicted bipartite signal. One conclusion which cannot be ruled out by these data is that these upstream basic amino acids could constitute a novel NLS, with a requirement for the predicted NLS in an auxiliary capacity. To hold true, this interpretation requires all four PSORTII-predicted NLSs to be non-functional and the new putative NLS formed by these amino acids to be conserved across kingdoms and recognized by all cells in which *piggyBac *functions. The role of NLSs can be influenced by proximal amino acids or tertiary configurations. Therefore, a simpler interpretation of these data is that one or more of the four predicted NLSs is functional and the identified upstream arginines are required for their activity.

Finally, sequencing analysis reveals the presence of an overlapping ZnF motif. When taken in the context of previous studies [53] this co-localization suggests the putative ZnF motif may constitute the *piggyBac *DNA binding domain. This is a logical arrangement when considered in the context of TPase evolution: allowing a TPase to carry out excision and reinsertion in the nucleus even while its sequence recognition sites are changing, giving rise to new family members. There is also the possibility that the ZnF may not function in DNA binding at all, but may be responsible for protein-protein interactions such as dimerization of the *piggyBac *TPase, binding host auxiliary factors, or heterochromatin interactions. Further investigation into this ZnF will need to be performed to elucidate its exact function, if any, in *piggyBac *transposition.

## Methods

### Plasmid construction

The EYFP open reading frame was obtained through PCR amplification of pXL-Bac-EYFP [64] using *Pfx *high-fidelity polymerase (Invitrogen, Carlsbad, CA) with primers supplying *Eco*RI (Note: all restriction enzymes obtained from New England Biolabs, Ipswich, MA) and *Not*I restriction sites at the 5' and 3' ends, respectively (table 1). The resulting PCR product was band isolated from a 9% agarose TAE gel, purified with QIAquick Gel Extraction columns (Qiagen, Valencia, CA) and digested with *Not*I and *Eco*RI. The inducible *D. melanogaster *metallothionein promoter vector pMT/V5-HisA (Invitrogen) was digested with the restriction enzymes *Not*I and *Eco*RI and treated with calf intestine alkaline phosphatase. The EYFP open reading frame was subsequently ligated into this vector to obtain pMT/EYFP. Sequencing and restriction analysis of the plasmid verified the presence and integrity of the EYFP open reading frame in pMT/EYFP. Functional fluorescence was tested by transient transfection of S2 cells with Cellfectin (Invitrogen) according to manufacturer's protocol. Expression of EYFP was induced by addition of CuSO_4 _(Sigma-Aldrich, St Louis, MO) to the medium at a final concentration of 500 μM. Fluorescence was observed with a Nikon Diaphot microscope.

**Table 1:** Primers and oligos used in this study

	Primer 1	Primer 2
pMT/EYFP	ACTGGAATTCATGGTGAGCAAGGGCGAGGAGCTG	CTAGAGTCGCGGCCGCTTTACTTGTA
pMT/pBac-EYFP	TAGAATTCTCGTGACTAATATATAATAAAATGGGT	ATTAGTGAATTCGAAACAACTTTGGCACATATC
pMT/NLS-1	AAGAATTCGGGATGGCTTCAAAGTCCACGAGGCGTAGC	ATTAGTGAATTCGAAACAACTTTGGCACATATC
pMT/NLS-2	CAGAATTCGTCATGGATCGATCTTTGTCAATGGTGTA	ATTAGTGAATTCGAAACAACTTTGGCACATATC
pMT/NLS-3	TGGAATTCAACATGCGTACGAAGTATATGATAAATGGA	ATTAGTGAATTCGAAACAACTTTGGCACATATC
pMT/NLS-4	TTGAATTCAACATGGCCCTTACTCTCGTCTCATATAAA	ATTAGTGAATTCGAAACAACTTTGGCACATATC
pMT/NLS-5	AGGAATTCAGTATGGAAAAATTTATGAGAAACCTTTAC	ATTAGTGAATTCGAAACAACTTTGGCACATATC
pMT/NLS-6	TAGAATTCTCGTGACTAATATATAATAAAATGGGT	CGGAATTCAACCTTTTCTCCCTTGCTACTGAC
pMT/NLS-7	TAGAATTCTCGTGACTAATATATAATAAAATGGGT	AGGAATTCGGGTCCGTCAAAACAAAACATCG
pMT/NLS-8	TAGAATTCTCGTGACTAATATATAATAAAATGGGT	GTGAATTCGTCACACATCATGAGGATTTTTAT
pMT/NLS-9	TAGAATTCTCGTGACTAATATATAATAAAATGGGT	AGGAATTCTGTGGACATGTGGTTATCTTTTCT
pMT/NLS-10	TAGAATTCTCGTGACTAATATATAATAAAATGGGT	GTGAATTCTGAAGTTGACCAACAATGTTTATT
pMT/NLS-11	ATATGGATCCGCATCGTGCAAAAAATGCAAAAAAGTT	TTTGGATCCCTCTTCAGTACTGTCATCTGATGTACC
pMT/NLS-13	TTTGGATCCATTTGCCTTTCGCCTTATTTTAGAGGGGC	AAAGGATCCGAAATGGTGAGCAAGGGCGAGGAGCTG
pMT/NLS-14	CCCGGATCCAACCTTTTCTCCCTTGCTACTGACATTATGGC	CCCGGATCCCCAGTAATGAAAAAACGTACTTACTGTACTTACTGCCCC
pMT/NLS-15	TTTTGAGCTCAACCTTTTCTCCCTTGCTACTGACATTATGGC	TTTTGAGCTCCCTACTTTGAAGAGATATTTGCGCGAT
pMT/NLS-16	TTTTGAGCTCAACCTTTTCTCCCTTGCTACTGACATTATGGC	TTTTGAGCTCCCAAATGAAGTGCCTGGTACATCAGATG
pMT/NLS-17	TTTTGAGCTCAACCTTTTCTCCCTTGCTACTGACATTATGGC	TTTTGAGCTCCCTACTTTGAAGGCCTATTTGGCCGATAATATCTCTAATATTTTG
pMT/NLS-18	TTTTGAGCTCAACCTTTTCTCCCTTGCTACTGACATTATGGC	TTTTGAGCTCCCTACTTTGGCCGCTTATTTGGCCGATAATATCTCTAATATTTTG
pMT/NLS-12	AATTCGTAATGGGGCCAGTAATGAAAAAACGTACTTACTGTACTTACTGCCCCTCTAAAATAAGGCGAAAGGCAAATG	
	AATTCATTTGCCTTTCGCCTTATTTTAGAGGGGCAGTAAGTACAGTAAGTACGTTTTTTCATTACTGGCGCCATTACG	

Primers used to make each of the vectors described in the text. Vector names are listed on the left with the cooresponding primers used to make the vector given on the right. In the case of pMT/NLS-12, ordered oligos were used as part of the final vector and not for PCR priming, as described in materials and methods.

The native *piggyBac *open reading frame sequence was PCR amplified from p3E1.2 [1] with end specific primers supplying an *Eco*RI site at either end (table 1). The PCR product was band isolated in 9% agarose TAE gel and digested with *Eco*RI. The vector pMT/EYFP was linearized with *Eco*RI, treated with calf intestine alkaline phosphatase and ligated to the *piggyBac *open reading frame to create a fusion consisting of the full length *piggyBac *open reading frame joined on its C-terminus to EYFP to form pMT/pBac-EYFP.

The vector pMT/EYFP was cut with *Eco*RI and treated with calf intestine alkaline phosphatase in preparation for the insertion of *piggyBac *sequences. The C-terminal *piggyBac *open reading frame truncations pMT/NLS-1 through pMT/NLS-5 (Δ1–100, Δ1–200, Δ1–302, Δ1–400, and Δ1–500, respectively) were all obtained by PCR amplification of p3E1.2 with *Pfx *high-fidelity polymerase, using a forward primer specific for the start of the *piggyBac *open reading frame and a reverse primer specific for each truncation as listed in table 1. The N-terminal truncations pMT/NLS-6 through pMT/NLS-10 (Δ497–594, Δ401–594, Δ301–594, Δ197–594, and Δ102–594, respectively) were also PCR amplified using a forward primer specific for each truncation (table 1) and a reverse primer specific for the end of the *piggyBac *open reading frame minus the stop codon.

The PCR product bands were isolated by 9% agarose TAE gel electrophoresis, cut with *Eco*RI and ligated into the prepared pMT/EYFP vector to obtain a chimeric open reading frame consisting of the *piggyBac *insertions fused upstream and in-frame with the downstream EYFP. Sequencing and restriction analysis verified the resulting ligations.

To obtain the deletion mutations pMT/NLS-11 (Δ551–571), pMT/NLS-13 (Δ572–594), and pMT/NLS-14 (Δ497–550), pMT/pBac-EYFP was PCR amplified using *Pfx *high-fidelity polymerase with inverted primers as noted in table 1. Briefly, pMT/pBac-EYFP was isolated from the dam methylating bacteria DH10B and subsequently used as a template. The majority of the plasmid except the deleted section was amplified and the resulting PCR reaction ethanol precipitated, washed with 70% ethanol, and resuspended in nuclease free water. Following resuspension, the DNA was cut with *Bam*HI to prepare the product ends for ligation, and *Dpn*I to digest the template. After a second ethanol precipitation, 70% ethanol wash, and resuspension in water, the PCR product was subject to self-ligation to form the respective plasmids. Restriction analysis and sequencing confirmed the integrity of the plasmids.

The deletions pMT/NLS-15 (Δ497–522, Δ572–594) and pMT/NLS-16 (Δ497–536, Δ572–594) were created by PCR amplification of dam methylated pMT/NLS-13 with inverted primers containing *Sac*I restriction sites at the 5' ends (table 1). The PCR products were ethanol precipitated, washed with 70% ethanol, and resuspended in nuclease free water. Following resuspension, the DNA was cut with *Sac*I to prepare the product ends for ligation and *Dpn*I to digest the template. After a second ethanol precipitation, 70% ethanol wash, and nuclease free water resuspension, the PCR product was subject to self-ligation to form the respective plasmids. Restriction analysis and sequencing confirmed the integrity of the plasmids.

The plasmids containing the amino acid substitutions, pMT/NLS-17 (Δ497–522, Δ572–594, K525A, R526A, R529A) and pMT/NLS-18 (Δ497–522, Δ572–594, R526A, R529A) within the pMT/NLS-15 deletion construct were made by PCR amplification of dam methylated pMT/NLS-13 with inverted primers similar to the construction of the pMT/NLS-15 deletion vector (table 1) Each ligation resulted in a plasmid containing the Δ497–522, Δ572–594 deletion open reading frame with the amino acid substitutions R526A, R529A and K525A, R526A, R529A respectively.

The pMT/NLS-12 (Δ1–550, Δ572–594) (fig. 3) fusion vector was constructed by annealing two oligonucleotides (table 1), to form a short double stranded DNA segment corresponding to the upstream and downstream outer boundaries of the PSORTII-predicted nuclear localization signal with *Eco*RI sticky ends. Briefly, 400 pmol of each oligo were combined in a total volume of 10 μl in a thin-walled PCR tube and heated by floating in 400 mL of boiling water. The water and oligos were then allowed to cool to room temperature undisturbed to facilitate annealing of the two oligos, keeping hairpinning and non-specific binding to a minimum. pMT/EYFP was then cut with *Eco*RI but not phosphatase treated. Combined with a large molar excess of the oligo mixture and ligated, the resulting vector was designated pMT/NLS-12.

### Cell culture and transfection

*D. melanogaster *Schneider 2 (S2) cells were grown in Schneider's medium (Gibco, Carlsbad, CA) supplemented with 10% FBS, 1 mg/mL streptomycin, and 25 μg/mL amphotericin at 28 degrees. Cells were transfected with Cellfectin (Invitrogen) using the recommended manufacturer protocol. Briefly, sterile coverslips were placed in the bottom of 9.4 cm^2 ^wells and used as the surface for cell adherence. 1 ml of cells were seeded in at 6 × 10^6^/ml in S2 medium supplemented with 10% FBS, 1 mg/mL streptomycin, and 25 μg/mL amphotericin (Sigma-Aldrich). The cells were allowed to adhere to the coverslip for 3 hours before undergoing transfection. Adherent cells were washed twice with serum-free S2 medium and resuspended in 800 μl serum-free S2 medium. For each transfection, 3 μl of Cellfectin was hydrated for 15 minutes in 97 μl sterile nuclease free water and added to 5 μg of DNA in 100 μl nuclease free water for a total volume of 200 μl. The Cellfectin-DNA mixture was allowed to incubate at room temperature for 20 minutes and added directly to the cells in a drop-wise manner followed by agitation to mix. The cells were incubated 18 hours at 28 degrees then given fresh S2 medium supplemented with 10% FBS, 1 mg/mL streptomycin, and 25 μg/mL amphotericin, as well as 500 μM CuSO_4 _(final concentration) to induce metallothionein promoter activity. Initial EYFP fluorescence was detectable at 4 hours post-induction through an EYFP filter (Chroma Technology Corp cat #40128; Excitation: 500 nm; Emission: 535 nm, Rockingham, VT) on a Nikon Diaphot (Nikon, Melville, NY) inverted phase contrast microscope, however the cells were analyzed at 48 h to allow for maximum EYFP signal.

### Confocal imaging

To prepare cells for confocal imaging, cells were transfected on coverslips as described above. At 48 hours post-induction, the media was aspirated from the coverslip, 200 μl of a 10 μM Draq5 (Biostatus Ltd., Leicestershire, UK) solution in 1× PBS was placed on the coverslip and incubated at room temperature for 10 minutes. The coverslips were then rinsed gently with 1× PBS and a slide was prepared with one drop of ProLong Gold antifade reagent (Invitrogen). The coverslip was sealed to the slide with nail lacquer and imaged with a Leica TCS SP2 True Confocal Scanner (Leica Microsystems, Bannockburn, IL) confocal microscope for EYFP and Draq5 fluorescence. Digital images represent 6 line averages and are cropped but otherwise remain unprocessed in the final images for publication.

## Authors' contributions

JHK created all plasmids used in this study, performed the confocal imaging, and prepared the manuscript. TSF performed all transfections and slide preparations. MJF conceived of the study and provided guidance. Special thanks to William Archer and Dr. Edward Hinchcliffe for their instruction in the use of the confocal microscope. All authors provided intellectual contributions as the study unfolded and reviewed the manuscript prior to submission.
